# Niacin Reverses Migratory Macrophage Foam Cell Arrest Mediated by oxLDL In Vitro

**DOI:** 10.1371/journal.pone.0114643

**Published:** 2014-12-18

**Authors:** Hua Huang, Pirkko Koelle, Markus Fendler, Angelika Schroettle, Michael Czihal, Ulrich Hoffmann, Peter Jan Kuhlencordt

**Affiliations:** Division of Vascular Medicine, Medical Clinic and Policlinic IV, University Hospital Munich, Munich, Germany; University Hospital Medical Centre, Germany

## Abstract

**Introduction:**

Niacin reduces vascular oxidative stress and down regulates inducible nitric oxide synthase, an enzyme mediating proatherosclerotic effects in part by increasing oxidative stress. Here, we evaluate whether Niacin reverses the redox sensitive migratory arrest of macrophages in response to oxidised(ox) LDL uptake.

**Material and Methods:**

Migration of RAW264.7 cells, a murine macrophage cell line and bone marrow derived macrophages from wildtype and iNOS knockout mice was quantified using a modified Boyden chamber. Unstimulated cells or cells preincubated with oxLDL or non-oxidised (n)LDL were treated with Nicotinic acid or Nicotinamide. Nitric oxide, peroxynitrite and ROS production were assessed using electron paramagnetic resonance (ESR). Additionally, flow cytometry analysis of apoptosis, fokal adhesion kinase (FAK), phalloidin, CD36, F4/80 macrophage marker and iNOS gene expression (PCR) were assessed.

**Results:**

Migration of Nicotinic acid, Nicotinamide treated cells or unstimulated cells did not differ (P>0.05). oxLDL treatment significantly reduced migration vs. unstimulated cells (p<0.05). In contrast, migratory arrest in response to oxLDL treatment was reversed by co-incubation with Nicotinic acid and Nicotinamide. The oxLDL-induced peroxynitrite formation in RAW264.7 cells was abolished by Niacin and glutathion (GSH) oxidation was significantly reduced. However, nitric oxide (NO)- and reactive oxygen species (ROS) production induced by oxLDL were not affected by Niacin treatment of RAW264.7 cells. In addition, Nicotinic acid and Nicotinamide reduced actin polymerization, a marker for migratory arrest.

**Discussion:**

Our data shows that oxLDL induced inhibition of macrophage migration in vitro can be reversed by Niacin. Furthermore, Niacin reduces peroxynitite formation and improves antioxidant GSH.

## Introduction

Niacin referring to Nicotinic acid and Nicotinamide has been used for almost sixty years to treat dyslipidemia in order to reduce/prevent atherosclerosis. As such Niacin markedly reduces plasma triglycerides, LDL-cholesterol, lipoprotein a, fibrinogen, plasminogen activator inhibitor-1 and increases HDL-C [1]. In the ARBITER 2 study Niacin in combination with statins slowed the progression of CAD and reduced cardiovascular events, an observation also made in several smaller studies [2], [3]. While most of the antiatherosclerotic effects are believed to result from its lipid modifying activity some evidence suggests that Nicotinic acid also reduces cardiovascular mortality independent from its lipid modifying properties [4]. In this respect, Niacin reduces plaque development independent of lipid lowering or HDL elevation in LDL receptor knockout mice [5]. In contrast, Niacin reduces atherosclerosis in ApoE*3Leiden.CETP mice, a model closely resembling human lipoprotein metabolism, mainly by reducing non HDL cholesterol [6]. Despite these positive results larger clinical trials like HPS2-THRIVE failed to show an additional risk reduction when Niacin/Laropiprant was given to patients already reaching target cholesterol levels with statin treatment [7]. Furthermore, AIM-HIGH was stopped prematurely because of a lack of benefit of Niacin [8]. Several reasons warrant further elucidation of these seemingly discrepant results, i.e. patients who reach target lipid levels have a relevant residual risk for adverse cardiovascular outcomes. Additionally, high risk patients intolerant to statins verify the need for alternative lipid lowering medications.

Nicotinamide, the metabolite of Nicotinic acid, also influences oxidative stress and has broad activities on many cell types, including regulation of cell adhesion, polarity, migration, proliferation and differentiation [9], [10]. Interestingly, Niacin also downregulated the expression of the inducible nitric oxide synthase in adipocytes, an enzyme expressed in atherosclerotic lesions which is capable of simultaneous generation of high concentrations of nitric oxide and superoxide. iNOS is not found in healthy vessels, however, in the microenvironment of inflammatory atherosclerotic lesions iNOS is expressed by macrophage/foam cells and vascular smooth muscle cells [11], [12]. The expression of inducible nitric oxide synthase (iNOS) in early and advanced atherosclerotic human and murine plaques may modulate cellular and molecular mechanisms that initiate and propagate atherosclerosis [13], [14]. Our previous research has shown that iNOS increases plaque development and lipid peroxides in atherosclerotic apoE knockout mice [15]. Furthermore, our past results have shown that iNOS simultaneously increases NO and O_2_- production and nitrosative/oxidative stress in the atherosclerotic plaques [16]. Changes in oxidative stress are associated with changes in macrophage/foam cell mobility [17] and recently published data from our laboratory suggests that nitrosative stress and subsequent lipid-peroxide formation are involved in trapping macrophage foam cells in the plaque [18]. Therefore, we here evaluate the role of Nicotinic acid and Nicotinamide in reversing the migratory arrest of macrophages in response to oxLDL uptake of RAW264.7 cells, a well charaterised model of macrophage foam cells as well as in primary bonemarrow cells of wild type and iNOS ko mice.

## Materials and Methods

### Reagents and antibodies

Nicotinic acid (N0761), Nicotinamide (N0636), NAC (A9165) and MCP-1, were obtained from Sigma. nLDL was obtained from Applichem (A6961). Dil-oxLDL was obtained from KALEN (770232-9). oxLDL was generated by incubating nLDL with 10 µM CuSO4 in PBS for 18 hours at 37°C [19]. For veryfication of LDL oxidation we performed an MDA-TBA HPLC assay and documented a significant increase in malondialdehyde (data not shown) signal [20]. The FITC-Annexin apoptosis kit was obtained from Becton Dickinson (556570). Antibodies used were directed towards following antigens: Nitrotyrosine (9691, Cell Signaling), FITC-phalloidin (P5282, Sigma), FAK (sc-932, Santa Cruz), iNOS (sc-650, Santa Cruz), β-Actin (sc-130656, Santa Cruz), APC-F4/80 (17-4801-82, eBioscience), FITC-CD36 (ab92560, abcam) and p-FAK (ab76244, abcam).

### Animals

Mice were backcrossed for 10 generations to the C57BL/6J genetic background. iNOS knockout (ko) mice were obtained from The Jackson Laboratories. All procedures performed were approved by the ethics committee of the medical faculty of the Ludwig-Maximilians-Universität München and the administration of Oberbayern (Approval No. 55.2-1-54-2532.3-67-12).

### Preparation of bone marrow cell derived macrophages (BMC)s

For BMC isolation, C57BL/6J and iNOS ko mice (8 weeks old, male) were euthanised by cervical dislocation and the femur and tibial bones were removed. BMCs were flushed with Dulbecco's Modified Eagle Medium (DMEM, 31966-021, Gibco) from the medullary cavities using a 25-G needle. Then, cells were cultured in DMEM medium supplemented with 10% fetal bovine serum, 1% penicillin/streptomycin in a 75 cm^2^ culture flasks (3290, Corning) and incubated at 37°C, 5% CO_2_. Cells from every two mice were cultured in one 75 cm^2^ culture flasks.

After 24 hours, 20 ng/ml mouse macrophage colony stimulating factor (M-CSF Mouse, CS-C2038, Cellsystems) was added into medium.

### Cell Culture

The murine macrophage cell line, RAW264.7 (CLS), was cultured and propagated in RPMI 1640 medium (31870-074, Gibco) supplemented with 10% fetal bovine serum (10500-064, Gibco), 2 mM L-glutamine (Biochrom) and 1% penicillin/streptomycin (Biochrom) in a 75 cm^2^ culture flasks at 37°C in humidified atmosphere containing 5% CO2 and air.

### In vitro migration assay (modified Boyden chamber migration assay)

Cell migration was assessed in a modified Boyden chamber using trans-well inserts with a 5 µm porous membrane (3421, Corning). Cells were first pre-treated with nLDL (100 µg/ml), oxLDL (100 µg/ml), Nicotinic acid (1 mM, 3 mM) or Nicotinamide (1 mM, 3 mM) at 37°C in 5% CO_2_ for 24 hours. Cells were then washed twice with PBS. Fifty thousand cells per group were added to 100 µl medium and introduced into the upper compartment of the trans-well. Into the lower compartment of the Boyden chamber 600 µl medium containing MCP-1 in a concentration of 10 ng/ml was added. The chambers were incubated at 37°C in 5% CO_2_ for 18 hours. During this time cells in the upper compartment were allowed to migrate to the lower side of the membrane. Membrane were harvested and cells on the lower side of the membrane fixed with methanol for 8 minutes and then stained with 1 drop of DAPI (Vector). At last, the lower side of the membrane was photographed using a fluorescence microscope with a ×10 objective. The number of the cells per picture were counted by Image Pro-Plus v.4.5 (The Proven Solution).

### Flow cytometry assays

To quantify apoptosis following oxLDL and Niacin treatment cells were washed twice with PBS. Then 10^5^ cells were suspend with 1×binding buffer incubated with FITC–annexin V and propidium Iodine (PI) at room temperature for 15 minutes in the dark, and then analyzed.

To measure the uptake of oxLDL, RAW264.7 cells were incubated with Dil-oxLDL (20 µg/ml) or Dil-oxLDL plus Nicotinic acid or Nicotinamide at 37°C in 5% CO_2_ for 2 hours. After that cells were washed twice by PBS and fluorescence intensity was quantified.

To measure polymerised actin (F-actin), RAW264.7 cells were pre-incubated with nLDL, oxLDL or oxLDL plus Nicotinic acid or Nicotinamide at 37°C in 5% CO_2_ for 24 hours. Following incubation, cells were washed twice with PBS, and 10^5^ cells were suspended in FACS buffer (PBS with 1% BSA) and fixed in 4% paraformaldehyde for 8 minutes. Cells were washed twice, treated with 0.1% Triton and stained with FITC-conjugated phalloidin at room temperature for 60 minutes in the dark and fluorescence intensity was quantified.

To measure phosphorylation of focal adhesion kinase (p-FAK), RAW264.7 cells were pre-incubated with nLDL, oxLDL or oxLDL plus Nicotinic acid or Nicotinamide at 37°C in 5% CO2 for 24 hours. Following incubation, cells were washed twice with PBS, and 10^5^ cells were suspended in FACS buffer (PBS with 1% BSA), fixed with 4% paraformaldehyde for 8 minutes, treated with 0.1% Triton and then incubated with anti-p-FAK antibody at room temperature for 40 minutes in the dark. Then cells were incubated with Alexa Fluor 488 conjugated secondary antibody (ab150077, abcam) at room temperature for 40 minutes in the dark, washed twice and fluorescence intensity was quantified.

For F4/80 and CD36 staining of BMCs 10^5^ cells were suspended in FACS buffer, followed by incubation with APC–F4/80 antibody at room temperature for 60 minutes in the dark. Subsequently, cells were washed twice with PBS and fluorescence intensity was quantified.

All flow cytometry was performed using a BD FACScan (Calibur). Data were analyzed by Flowjo 7.6.1.

### Real time PCR

Total RNA was extracted from cells using TriFast reagent (30-2010, peqGOLD). cDNA was synthesized from 1 µg of total RNA using iScript cDNA synthesis kit (170-8890, Bio-Rad) following the manufacturer's instructions. Gene expression was analyzed by quantitative RT-PCR using the EVA Green PCR Master MIX (172-5201, Bio-Rad) with the Mx3000P detection system and relative quantification software (Stratagene). The expression levels of each gene were determined using the comparative Ct method and normalized to β-actin, as internal control. The primers are listed in Table 1.

**Table 1:** Primers used for real-time PCR analysis.

Gene	Primer sequence	
	Forward	Reverse
iNOS	5′-GTTTCTGGCAGCAGCGGCTC-3′	5′-GCTCCTCGCTCAAGTTCAGC-3′
β-actin	5′-CGTGGGCCGCCCTAGGCACCAGGG-3′	5′-GGGAGGAAGAGGATGCGGCAGTGG-3′
t-FAK	5′-GAGAATCCAGCTTTGGCTGTT-3′	5′-GGCTTCTTGAAGGAACTTCT-3′

### Western blot

RAW264.7 cells were lysed using RIPA buffer (89900, Thermo). Lysates were separated by SDS-PAGE, transferred to PVDF membranes and probed with antibodies for β-Actin (1∶500 diluted), 3-nitrotyrosine (1∶200 diluted), iNOS (1∶200 diluted) and p-FAK (1∶1000 diluted), followed by chemiluminescence detection. Band intensities were quantified using Image J. Immunoprecipitations were performed following the Immunoprecipitation Kit's instructions (10006D, novex).

### HPLC

Cells were washed twice with PBS and treated on ice with tri-n-butylphosphine in dimethylformamide for 30 min at 4°C. After that cells were scraped and sonicated and GSH and GSSG were measured and calculated respectively by HPLC as described [21].

### Measurement of NO Production by Electron Spin Resonance (ESR)

NO production of RAW264.7 cells was measured using iron-diethyldithiocarbamate (Fe(DETC)_2_) as a spin trap and ESR detection. Briefly, cells were washed twice by PBS and suspended in 4°C chilled Krebs-Hepes Buffer (KHB). 10 ml FeSO4 and 10 ml DETC solutions were bubbled with N_2_ for 20 min, and each 250 µl were added to each dish (total amount 1 ml). NO produced by cells was captured by Fe-(DETC)_2_ for 1 hour in 37°C KHB. Cells were scraped and 100 µl of the cell suspension was frozen in liquid N_2_ and subject to ESR analysis. Following ESR measurement the protein concentration of each sample was quantified with a protein assay reagent (Bio-Rad) and used to normalize ESR signal intensity. ESR measurements were done using a bench top e-scan ESR spectroscope. The instrumental settings were listed in Table 2.

**Table 2:** Electron Spin Resonance settings for NO, ROS and peroxynitrite Measurement.

	Measurement of NO	Measurement of reactive oxygen species (ROS) and peroxynitrite
Centre field	3308 G	3388 G
Sweep width	80 G	132 G
Microwave frequency	9.495 GHz	9.497 GHz
Microwave power	50 mW	1.25 mW
Modulation Amplitude	4.6 G	1.63 G
Modulation frequency	86 kHz	86 kHz
Time constant	81.92 ms	40.96 ms
Time constant	20.48 ms	10.24 ms
Number of scans	30	50

### Measurement of reactive oxygen species (ROS) and peroxynitrite by ESR

The generation of ROS by RAW264.7 cells was measured by ESR detection (e-scan ESR spectrometer). A stock solution of spin probe (1-hydroxy-3-methoxycarbonyl-2,2,5,5-tetramethylpyrrolidine, CMH) (10 mM, 2.3 mg for 1 ml KHB) in buffer containing 25 µM Defferoxamine (DF) and 5 µM DETC was prepared. 10^6^ cells seeded in 6-wells were washed twice by PBS and then suspended in KHB containing 25 µM DF and 5 µM DETC at 4°C. 100 µL of cell suspension and 2.5 µL of spin probe solution were mixed. ROS production was assessed by incubating cells with CMH for 30 min at 37°C. The reaction was stopped on ice.

Uric acid (Sigma) was used as a scavenger for peroxynitrite [22]. Peroxynitrite formation of RAW264.7 cells was calculated as the difference of ROS production between each group before and following uric acid treatment. Following ESR measurement the protein concentration of each sample was quantified with a protein assay reagent (Bio-Rad) and used to normalize ESR signal intensity.

The instrumental settings were listed in Table 2.

### Statistics

Statistical analysis was performed using ANOVA. A P-value of less than 0.05 was considered significant. Data are expressed as mean±SD. The analyses were formed using SPSS Statistics 17.0.

## Results

### Nicotinic acid and Nicotinamide reverse oxLDL mediated migratory arrest of RAW264.7 cells

**Figure 1:**
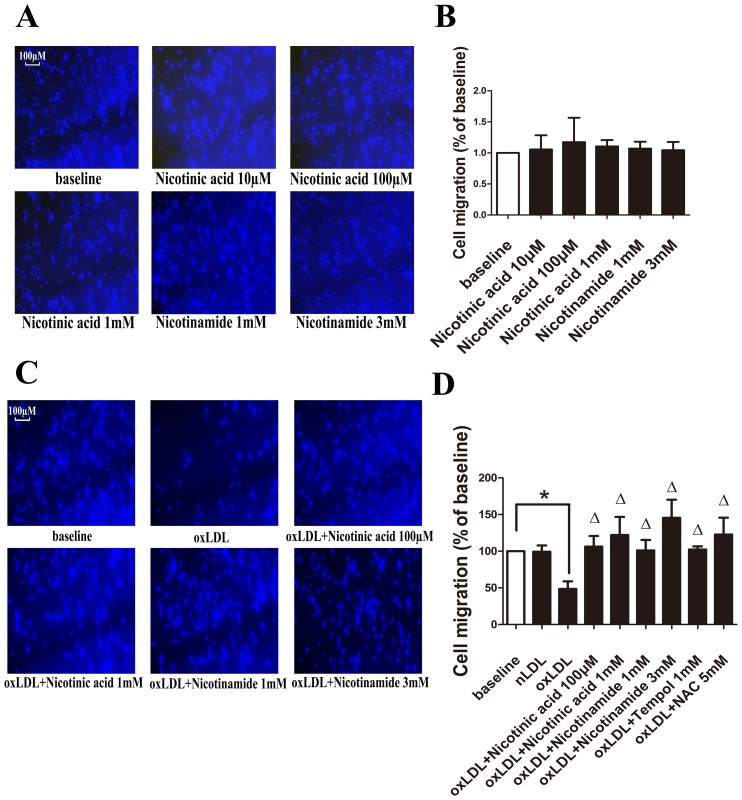
Migration assay of RAW264.7 cells. **A**. Migrated RAW264.7 cells on the lower side of the membrane were stained with DAPI and counted under a fluorescence microscope using a ×10 objective. Representitive pictures of each treatment group. **B**. Migrated cells of each group. The migration of cells between each group was not significantly different. (P>0.05, n = 5). **C**. Migrated RAW264.7 cells on the lower side of the membrane were stained with DAPI and counted under a fluorescence microscope using a ×10 objective. Representitive pictures of each treatment group. **D**. Migrated cells of each group. Significance was determined by ANOVA. (*, p<0.05, Δ, p<0.05 compared with oxLDL group, n = 5).

The migration of cells treated with Nicotinic acid or Nicotinamide only, compared to the unstimulated group did not significantly differ. (P>0.05) (Fig. 1A, 1B) oxLDL treatment significantly reduced macrophage migration compared to unstimulated cells (74.4±11.2 versus 179.1±81.5, p<0.05), while the migratory arrest in response to oxLDL treatment was reversed by coincubation with Nicotinic acid at the concentration of 1 mM (74.4±11.2 versus 196.2±65.6, p<0.05). Interestingly, the migratory arrest in response to oxLDL treatment was also reversed by coincubation with Nicotinamide with both concentration of 1 mM (74.4±11.2 versus 166.4±53.2 p<0.05) and 3 mM (74.4±11.2 versus 233.8±53.0, p<0.05).(Fig. 1C, 1D). Additionally, the ROS scavanger tempol and NAC were also able to reverse the migratory arrest induced by oxLDL(Fig. 1D).

### Nicotinic acid and Nicotinamide did not influence Apoptosis of RAW264.7 cells

To exclude the possibility that changes in migration were caused by differences in cell viability between the different treatment groups, we analyzed Annexin V and propidium lodine staining of the RAW264.7 cells by flow cytometry. All measurements were performed five times for each group. The percentage of dead cells (PI and Annexin V double positive cells) of each group were baseline (10±6.63), nLDL (14.7±2.99), oxLDL(13.3±2.54), oxLDL+Nictinic acid 100 µM (10.4±4.64), oxLDL+Nictinic acid 1 mM(11.1±4.32), oxLDL+Nicotinamide 1 mM (11.9±3.41), oxLDL+Nicotinamide 3 mM(10.3±2.81). There were no significant differences between the percentage of live cells, apoptotic cells and dead cells between groups.

### Nicotinic acid and Nicotinamide did not change oxLDL uptake and expression of F4/80 by RAW264.7 cells

**Figure 2:**
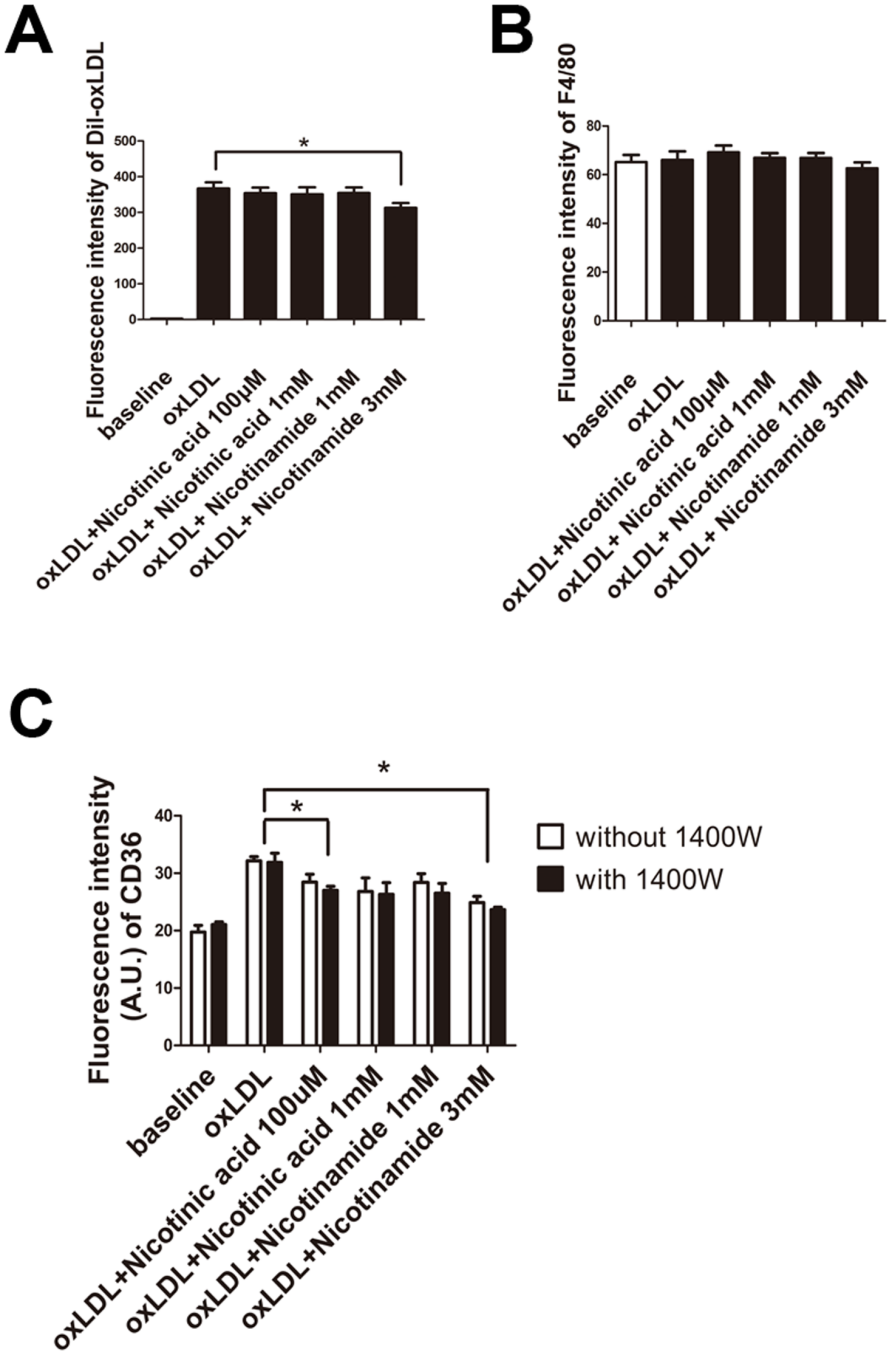
Flow cytometry of Dil-oxLDL, and F4/80. **A**. Cells were preincubated with Dil-oxLDL, then Nican in a concentration of 100 µM or 1 mM, or Nicotinamide in a concentration of 1 mM or 3 mM were added. The fluorescence intensity of Dil-oxLDL treated cells was significantly reduced when cell were treated with Nicotinamide at 3 mM. (*, P<0.05, n = 5). **B**. The fluorescence intensity of F4/80 of each group was not significantly different (P>0.05, n = 5). **C**. Fuorescence intensity of CD36 in each group. (*, P<0.05, n = 5).

To investigate whether the uptake of oxLDL by RAW264.7 cells changes following Nicotinic acid or Nicotinamide treatment we quantified fluorochrome labeled Dil-oxLDL uptake of cells by FACS. While in general no differences in LDL uptake were noted between groups, the highest dose of Nicotinamide (3 mM) significantly reduced oxLDL uptake of RAW264.7 cells. (Fig. 2A) Additionally, the expression of F4/80, a macrophage maker and CD36, a scavenger receptor of RAW264.7 cells were measured by flow cytometry. The expression of F4/80 did not differ between groups. (Fig. 2B) However, the fluorescence intensity of CD36 of oxLDL treated cells was significantly stonger compared to unstimulated cells, but was significantly reduced when cells were treated with Nicotinic acid 100 µM, Nicotinamide 1 mM and 3 mM (Fig. 2C).

### oxLDL induced iNOS Expression of RAW264.7 cells was reduced by Niacin

**Figure 3:**
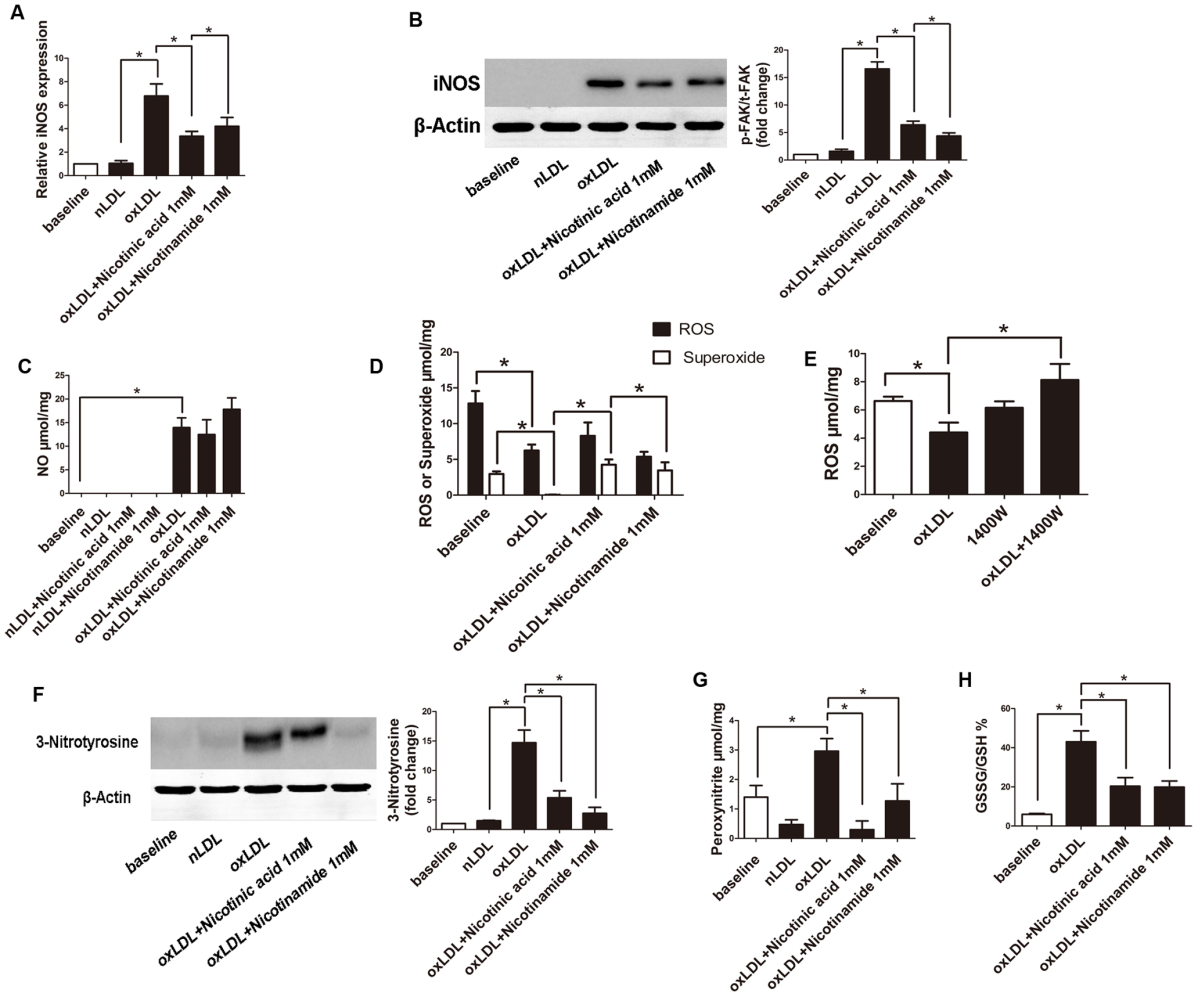
Measurement of iNOS, p-FAK, 3-Nitrotyrosine expression, production of NO, ROS, superoxide, peroxynitrite and GSSG/GSH in RAW264.7 cells. **A**. iNOS expression of RAW264.7 cells of each group was tested by “real time” PCR using comparative CT method. (*, P<0.05, n = 3). **B**. Representative western blot of iNOS expression of RAW264.7 cells of each group. (*, P<0.05, n = 3). **C**. The intracellular NO production was measured by ESR. (*, P<0.05, n = 5). **D**. Intracellular ROS and superoxide was measured by ESR. (*, P<0.05, n = 5). **E**. Intracellular ROS was measured by ESR. (*, P<0.05, n = 5). **F**. Representative western blot of 3-Nitrotyrosine. Band intensities were quantified by ImageJ and normalized to β-actin. (*, P<0.05, n = 3). **G**. Extracellular peroxynitrite was measured by ESR. (*, P<0.05, n = 6). **H**. GSSG/GSH of RAW264.7 cells measured by HPLC.(*, P<0.05, n = 4).

We used realtime PCR and western blot to see whether the iNOS expression differed between treated groups. (Fig. 3A and 3B) iNOS expression was increased following oxLDL treatment and significantly reduced by co-incubated with Niacin.

### Niacin leads to a reduction in peroxynitrite generation induced by oxLDL

With the co-incubation with Nicotinic acid and Nicotinamide, the oxLDL-induced peroxynitrite formation in RAW264.7 cells measured by ESR was abolished. Additionally, western blot analysis of 3-Nitrotyrosine, considered a footprint of peroxynitrite formation revealed similar results. However, NO- and ROS production induced by oxLDL were not affected by Nicotinic acid and Nicotinamide treatment of RAW264.7 cells, but decreased ROS production by oxLDL was reversed by 1400W, an iNOS inhibitor (Fig. 3C, 3D, 3E, 3F and 3G).

### oxLDL induced Glutathione disulfide (GSSG)/Glutathione (GSH) was reduced by Niacin

Antioxidant enzymes, such as glutathione peroxidases and peroxiredoxins, generate glutathione disulfide during the reduction of peroxides such as H_2_O_2_ and ROOH. Glutathione disulfide (GSSG)/Glutathione (GSH) was measured by HPLC. Incubation of cells with oxLDL increased GSSG, and this increase was reversed by co-incubation with Nicotinic acid and Nicotinamide (Fig. 3H).

### Nicotinic acid and Nicotinamide reverse oxLDL mediated migratory arrest of bone marrow cells

**Figure 4:**
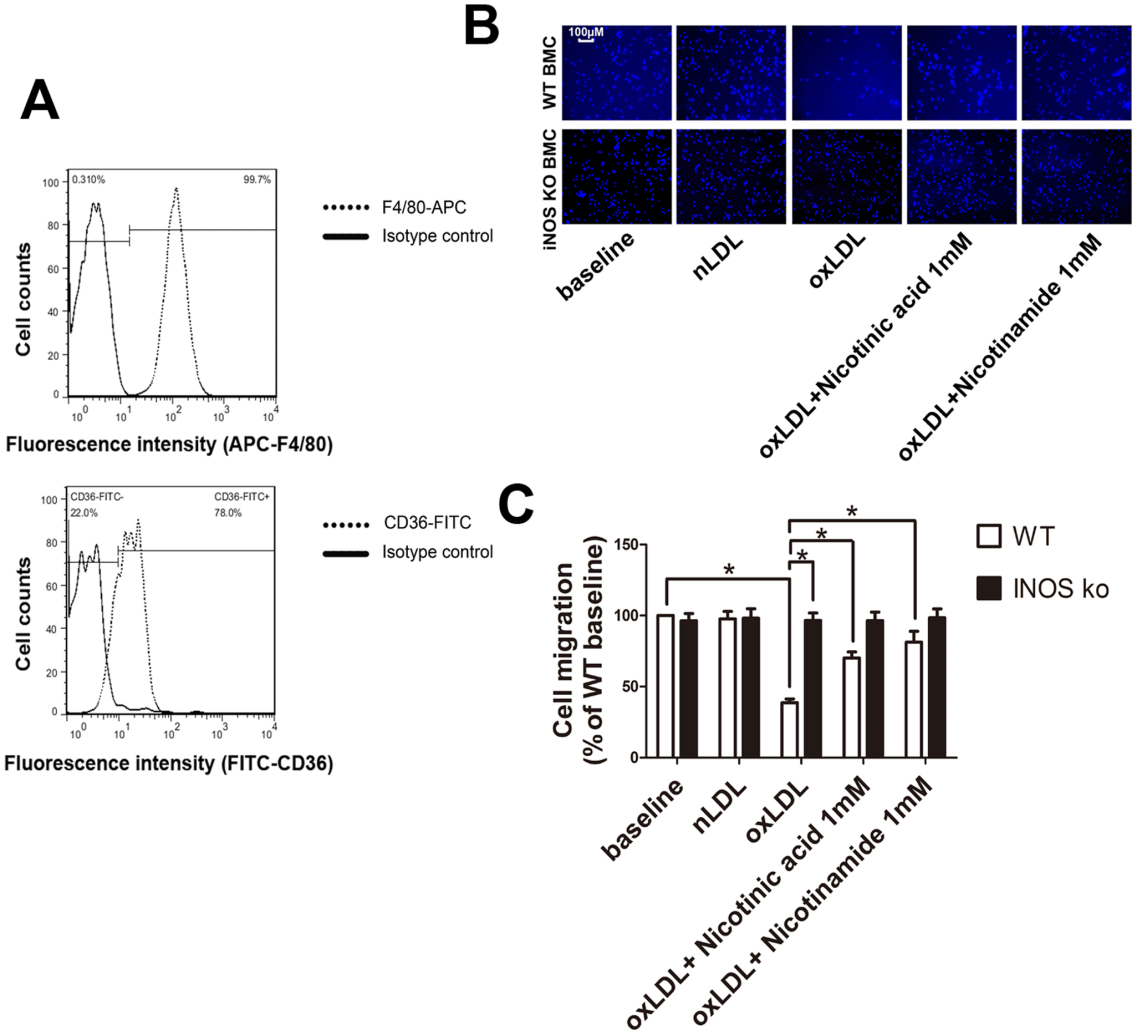
Migration assay of bone marrow derived macrophages. **A**. Bone marrow derived macrophages were stained with fluorochrome labeled anti-F4/80 and anti-CD36 antibodies. Fluorescence intensity was analysed by flow cytometry. **B**. Migrated RAW264.7 cells on the lower side of the membrane were stained with DAPI and counted under a fluorescence microscope using a ×10 objective. Representitive pictures of each treatment group. **C**. Migration of oxLDL treated cells was significantly reduced compared to unstimulated cells. Migration of oxLDL treated cells was significantly different compared to Nicotinic acid 1 mM, Nicotinamide 1 mM and 3 mM. (*, P<0.05, n = 5).

Here, we isolated bone marrow derived macrophages of iNOS knockout and wild type mice to test differences in cell migration in a model of chronic iNOS deficiency. To confirm that bone marrow derived cells were indeed macrophages, we stained the freshly isolated cells for F4/80 and CD36 and analyzed them by flow cytometry. (Fig. 4A) Under baseline conditions iNOS knockout macrophages migrated to the same extent as wild type macrophages. In contrast, in the presence of oxLDL, wild type macrophage migration was significantly inhibited, while iNOS knockout macrophage migration was not inhibited. Moreover, Nicotinic acid and Nicotinamide reversed migratory arrest in wild type macrophages, however, there was no additional gain in migration in the iNOS knockout groups (Fig. 4B, 4C).

### oxLDL induced focal adhesion kinase and actin polymerization were reduced by Niacin

**Figure 5:**
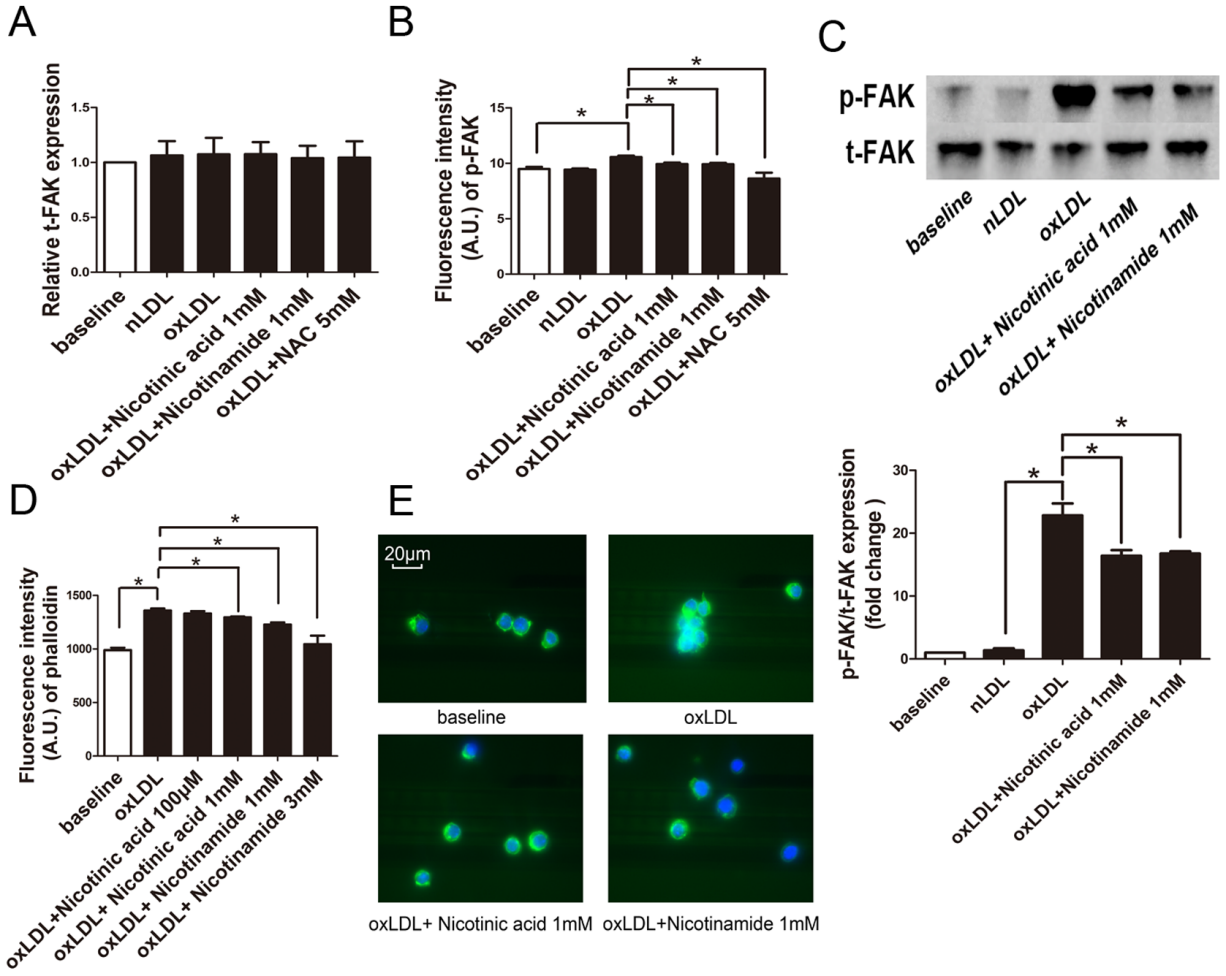
Expression of t-FAK and phalloidin in RAW264.7 cells. **A**. t-FAK expression of RAW264.7 cells of each group was tested by “real time” PCR using comparative CT method. (n = 5). **B**. The expression of p-FAK in RAW264.7 cells of each group was subjected to analysis by flow cytometry. (*, p<0.05, n = 5). **C**. Expression of p-FAK and t-FAK was measured by western blot. (*, P<0.05, n = 3). **D**. RAW264.7 cells were stained with FITC-phalloidin and subjected to analysis by flow cytometry.(n = 5). **E**. RAW264.7 cells of each group were stained with FITC-phalloidin and DAPI and then photographed under a fluorescence microscope using a ×63 objective.

As the inhibition of cell migration may be associated with enhancement of focal adhesion kinase (FAK) phosphorylation and actin polymerization we measured these markers. Real time PCR analysis revealed no difference in the expression of focal adhesion kinase (t-FAK) between groups (Fig. 5A).

But indeed, phosphorylation of the focal adhesion kinase (p-FAK) showed a significant increase of p-FAK in oxLDL treated RAW264.7 cells, an effect which was completely abolished by Nicotinic acid, Nicotinamide or NAC treatment by FACS analysis. (Fig. 5B) Moreover, additional western blot analysis for p-FAK/t-FAK showed simlar results (Fig. 5C).

FACS analysis showed that the amount of polymerized actin did not differ between baseline and nLDL treatment. However, oxLDL treatment increased phalloidin staining, an effect which was abrogated by Nicotinic acid and Nicotinamide (Fig. 5D, 5E).

## Discussion

In contrast to a large body of evidence regarding the mechanisms by which statins protect against cardiovascular disease, the cardioprotective mechanisms of Niacin are still not completely understood.

The water soluble vitamin Niacin (Vitamin B3) was originally described as an antiatherosclerotic drug due to its ability to reduce plasma non HDL lipids and increase HDL cholesterol [2]. Additional atheroprotective effects which are independent of lipid lowering involve modulation of oxidative stress [4] as well as broad activities on many cell types, including regulation of cell adhesion, polarity, migration, proliferation and differentiation [9], [10]. Although the recent large outcome trials AIM-HIGH and HPS2-THRIVE failed to reveal additional beneficial effects of Niacin on top of statin treatment in patients with on target LDL choleterol plasma concentrations [23], [24]. other studies have shown that nicotinic acid may have beneficial effects in acute and chronic vascular disease independently of its effects on plasma lipids [5], [25]. Moreover, Niacin reduces atherosclerosis in hyperlipidemic mice and experimental evidence suggest that the antiatherogenic effects involve modulation of lipid profiles as well as lipid independent mechanisms. Along these lines data indicates that Niacin inhibits vascular inflammation by decreasing endothelial ROS production, subsequent LDL oxidation and inflammatory cytokine release [4]. New strategies to reduce inflammation and increase reverse cholesterol transport by modulating immune cell signaling involved in the progression of atherosclerosis have been proposed as innovative approaches to prevent and treat atherosclerosis. Interestingly, Niacin was found to reduce iNOS expression in adipocytes [26]. Data from our lab revealed that iNOS inhibition in macrophage foam cells reduces nitrosative stress and lipid peroxide formation, which in turn restores migration in oxLDL fed cells [18].

Moreover, our previous research revealed reduced atherosclerotic plaque development upon reduction of nitrosative stress in atherosclerotic apoE knockout mice [13], [15]. Furthermore, our past results show that iNOS increases nitrosative stress in atherosclerotic plaques [16].

Here we demonstrate that Niacin reverses the migratiory arrest of macrophage foam cells by oxLDL uptake. Our data demonstrates that Niacin reduces oxLDL induced iNOS expression and nitrosative stress. Furthermore, we found that Niacin reduces actin polymerization and the phosphorylation of focal adhesion kinase (p-FAK). The latter finding is of significance since actin-based motility, driven by the assembly of actin filaments determines direct movement of cells. The cell migration process consists of actin polymerization driven lamellipodia extension, disruption of existing- and formation of new focal contacts [27]. Mechanistically, this inhibition was associated with enhancement of focal adhesion kinase (FAK) phosphorylation and actin polymerization [17], [28]. Since the signaling pathway required for actin polymerization includes activation of FAK [29], it has been suggested that the sustained activation of FAK [30] and its hyperphosphorylation is associated with the inhibition of cell migration [31].

In the current study we performed a detailed analysis of oxidative stress using ESR spectroscopy to measure reactive oxygen species (ROS), NO and peroxynitrite production by iNOS since oxidative stress may directly lead to vascular injury. In atherosclerotic lesions oxidative stress is increased compared to healthy vascular tissue, suggesting that oxidative stress is one important mediator or direct factor causing the trapping of macrophages in the intima [32]. In order to antagonize oxidative stress cells have developed potent antioxidative strategies. As such glutathione (GSH) is an important antioxidant in plants, animals, fungi and some bacteria and archaea, preventing damage to important cellular components caused by reactive oxygen species such as free radicals and peroxides [33]. Antioxidant enzymes, such as glutathione peroxidases and peroxiredoxins, generate glutathione disulfide (GSSG) during the reduction of peroxides like hydrogen peroxide (H_2_O_2_) and organic hydroperoxides (ROOH) [34]. Our results indicate that oxidation of intracellular GSH is reduced following Niacin treatment. Interestingly, total NO and ROS levels did not change following Niacin treatment.

Our study reveales that Niacin reversed the decrease of ROS- and specifically superoxide production caused by oxLDL uptake and reduced the oxLDL mediated increase of peroxynitrite formation in RAW264.7 cells as measured by ESR and western blot. oxLDL may have caused a decrease of superoxide concentration because superoxide reacted with NO to form peroxynitrite. The formation of peroxynitrite is a diffusion controlled reaction with a rate constant of 6,7×10^9^ mol-l s-1 between O_2_^−^ and NO, which is three times faster than enzymatic degradation of O_2_^−^ [35]. Our results are in line with this proposed biochemistry. Treatment of oxLDL fed RAW264.7 cells with the iNOS inhibitor W1400 leads to an increase of ROS production. Moreover, treatment of oxLDL fed RAW264.7 cells with SOD revealed that the O_2_^−^ signal vanished completely and previous publications indicate that high concentrations of O_2_^−^ react with NO to form peroxinitrite in a way completely consuming O_2_^−^ [36].

We found a difference in iNOS expression, however, changes in expression levels did not change enzyme activity to make a significant difference to NO production. A possible explanation is that aside from its transcriptional regulation, multiple post-translational modifications of iNOS have been identified that allow complex regulation of the catalytic activity of these enzymes [37]–[42].

The observation that Niacin inhibited the formation of peroxynitrite may render an explanation for our finding that net superoxide production of RAW264.7 cells was not reduced.

Recently published data from our laboratory showed that increased peroxynitrite formation leads to increased production of lipid peroxides [18], which are strong inducers of protein tyrosine phosphatases (PTPs) by their oxidation [43]. In this way, oxidative inactivation of PTP may result in sustained phosphorylation of FAK and increased actin polymerization, causing migratory arrest.. In agreement with this concept scavenging of ROS increased migration in oxLDL treated cells in our experiments. Interestingly, Nicotinamide, a metabolite of Niacin shows the same or an even stronger effect on reversing oxLDL mediated inhibition of cell migration.

In conclusion, our results reveal that oxLDL induced inhibition of macrophage migration may be reversed by Niacin, rendering a novel explanation for Niacin's atheroprotective effects on cardiovascular disease independent of its effects on plasma lipids. Furthermore, Niacin reduced peroxynitrite formation and GSH oxidation.
